# Supplementary material for: Assessing factors influencing students’ perceptions towards animal species conservation
Source: PeerJ. 2023 Jan 9;11:e14553. doi: 10.7717/peerj.14553 (PMC9835705; doi:10.7717/peerj.14553)
Supplement: Supplemental Information 2 [file peerj-11-14553-s002.docx]

PERCEPÇÃO SOBRE VERTEBRADOS SELVAGENS

PERFIL DO ENTREVISTADO

1. Termo de Consentimento Livre e esclarecido (TCLE)
2. Sua idade (somente números) (deve ser maior de idade)
3. Gênero: Masculino ( ) Feminino ( )
4. Curso -
5. Instituição -
6. Período que você está cursando:
7. Cidade -
8. Religião -
9. Renda Familiar -
10. Tem algum animal de estimação ou já teve? Se sim, qual o animal?

Em relação a cada animal abaixo, por favor, marque uma opção na escala correspondente ao lado, expressando sua relação com o mesmo. Para cada característica, exemplo: (Prejudicial, Útil, Bonito) atribuída ao animal, você deverá optar por uma opção. Exemplo: (Discordo muito, concordo um pouco, concordo muito)

11.

| **Morcego é um animal** | **Discordo muito** | **Discordo um pouco** | **Nem discordo nem concordo** | **Concordo um pouco** | **Concordo muito** |
| --- | --- | --- | --- | --- | --- |
| Perigoso |  |  |  |  |  |
| Útil |  |  |  |  |  |
| Feio |  |  |  |  |  |
| Inofensivo |  |  |  |  |  |
| Preservado |  |  |  |  |  |
| Bonito |  |  |  |  |  |
| Prejudicial |  |  |  |  |  |
| Não preservado |  |  |  |  |  |

** As próximas questões abordam os mesmos itens da escala acima para 17 espécies animais, seguindo o mesmo modelo, por exemplo “Tubarão é um animal, urubu é um animal...”
