# Supplementary material for: Assessing factors influencing students’ perceptions towards animal species conservation
Source: PeerJ. 2023 Jan 9;11:e14553. doi: 10.7717/peerj.14553 (PMC9835705; doi:10.7717/peerj.14553)
Supplement: Supplemental Information 3 [file peerj-11-14553-s003.docx]

**PERCEPTION ABOUT WILD VERTEBRATES**

INTERVIEWEE PROFILE

1. The Free and Clarified Consent Term

2. Your age (numbers only)

3. Gender: Male ( ) Female ( )

4. Course -

5. Institution -

6. Bachelor semester:

7. City -

8. Religion -

9. Family Income -

10. Do you have any pets or have you ever had them? If yes, which animal?

Guidance to answer the next questions:

For each animal below, please mark an option on the corresponding scale on the side, expressing your relationship with it. For each characteristic, eg: (Harmful, Useful, Beautiful) assigned to the animal, you must choose an option. Example: (Strongly Disagree, Agree Somewhat, Agree Strongly).

11.

| **Bat is an animal** | **I strongly disagree** | **I disagree a little** | **I neither disagree nor agree** | **I agree a little** | **I agree a lot** |
| --- | --- | --- | --- | --- | --- |
| Dangerous |  |  |  |  |  |
| Useful |  |  |  |  |  |
| Ugly |  |  |  |  |  |
| Harmless |  |  |  |  |  |
| Preserved |  |  |  |  |  |
| Beautiful |  |  |  |  |  |
| Harmful |  |  |  |  |  |
| Not preserved |  |  |  |  |  |

** The next questions address the same items from the scale above for 17 animal species, following the same model, for example “Shark is an animal, Vulture is an animal...”
